# Space-Time Statistical Insights about Geographic Variation in Lung Cancer Incidence Rates: Florida, USA, 2000–2011

**DOI:** 10.3390/ijerph15112406

**Published:** 2018-10-30

**Authors:** Lan Hu, Daniel A. Griffith, Yongwan Chun

**Affiliations:** School of Economic, Political and Policy Sciences, The University of Texas at Dallas, 800 West Campbell Road, Richardson, TX 75080, USA; dagriffith@utdallas.edu (D.A.G.); ywchun@utdallas.edu (Y.C.)

**Keywords:** lung cancer incidence, positive spatial autocorrelation, negative spatial autocorrelation, random effects, spatial autocorrelation mixture

## Abstract

The geographic distribution of lung cancer rates tends to vary across a geographic landscape, and covariates (e.g., smoking rates, demographic factors, socio-economic indicators) commonly are employed in spatial analysis to explain the spatial heterogeneity of these cancer rates. However, such cancer risk factors often are not available, and conventional statistical models are unable to fully capture hidden spatial effects in cancer rates. Introducing random effects in the model specifications can furnish an efficient approach to account for variations that are unexplained due to omitted variables. Especially, a random effects model can be effective for a phenomenon that is static over time. The goal of this paper is to investigate geographic variation in Florida lung cancer incidence data for the time period 2000–2011 using random effects models. In doing so, a Moran eigenvector spatial filtering technique is utilized, which can allow a decomposition of random effects into spatially structured (SSRE) and spatially unstructured (SURE) components. Analysis results confirm that random effects models capture a substantial amount of variation in the cancer data. Furthermore, the results suggest that spatial pattern in the cancer data displays a mixture of positive and negative spatial autocorrelation, although the global map pattern of the random effects term may appear random.

## 1. Introduction

Spatial scientists, practitioners, and policy makers are interested in understanding the spatial variation in cancer rates at various geographic scales and resolutions (e.g., [1]). One commonly employed geographic resolution is the county, because aggregating especially counts of rare cancer cases by county for ecological analyses almost always preserves patient confidentiality [2]. This ethical and legal goal is at the expense of data analysis accuracy and precision, as well as data analytic complications such as the ecological fallacy. Meanwhile, depicting finer geographic resolution rates with choropleth or kernel density smoothed maps can maintain patient confidentiality while improving data analysis accuracy and precision (e.g., [3]), and help avoid or minimize such complications as the ecological fallacy.

Often, one objective of such research is to investigate associations between cancer rates and socio-economic/demographic characteristics. The availability of such covariates, generally retrieved from government census publications, tends to be very limited for fine geographic resolutions (e.g., census blocks). Furthermore, as a rare event, many cancer rates are often zero in a sizeable number of areal units at a very fine geographic resolution. Because a spatial analysis of cancer rates defies conducting scientific human subject experiments on ethical grounds, and, hence, is observational/correlational in nature, researchers seldom have a priori information about covariates that would make significant contributions to the geographic variation in cancer rates. Quasi-experimental designs have uncovered selected surrogate covariates supportable by scientific rationales, such as income/poverty and access to cancer screening/diagnosis/treatment [4], age and increased risk of developing cancer [5], and education and lifestyle cancer-prevention behaviors [6].

A random effects (RE) model seeks to control for time-invariant unobserved heterogeneity in data. RE may be viewed as areal unit specific effects attributable to unknown latent variables. Although omitted variables influence a regression analysis of georeferenced cancer rates, introducing a RE term can account for some of their effects. With regard to RE, Besag et al. [7] specify that their geographic distributions comprise the following two components: spatially structured random effects (SSRE), and spatially unstructured random effects (SURE). A Bayesian hierarchical model, in which a prior distribution can substitute for repeated measures in a space-time series, can be specified to estimate both SSRE and SURE terms. In this Bayesian context, a SSRE component often is modelled with a conditional autoregressive (CAR) specification that captures spatial autocorrelation (SA) (SA refers to Tobler’s first law of geography: everything is related to everything else, but nearby phenomena are more related than distant phenomena. In other words, either similar (positive SA) or dissimilar (negative SA) attribute values tend to cluster together on a map. Georeferenced data rarely are void of map pattern (i.e., have no SA)) latent in georeferenced data, and a SURE component is specified with an independent normal distribution. In this specification, SA is accounted for directly through parameters. In contrast, for space-time data, a second-order model is specified in which SA is accounted for through correlations among observations. That is, repeated measures are furnished for each spatial unit over time in a space-time data series. In this paper, an estimated RE term represents omitted variables, indicating that geographic distributions of cancer rates contain a considerable amount of unexplained variation, particularly at the census tract resolution. This cancer data expectation is attributable to the lengthy exposure lag that characterizes many cancers (i.e., a cancer being triggered long before its actual diagnosis), combined with the movement of people, and the departure of carcinogenic sources over time.

The specification of [7] addresses only positive SA (PSA) situations. More recently accumulated empirical evidence indicates that a number of georeferenced phenomena seemingly exhibiting (near-) zero SA actually contain a mixture of PSA and negative SA (NSA) as two compensating components [8]. PSA arises from cooperative processes that involve intensifying spatial externalities, whereas NSA arises from competitive processes that involve abating spatial externalities. In this paper, a SSRE term consistently decomposes into a PSA-NSA mixture, which is barely investigated in the literature. Possible reasons for this mixed spatial pattern in cancer data include: (1) that geographic distributions of cancer cases display some degrees of global, regional, and local map patterns, which potentially arise from a collocation of similar socio-economic/demographic characteristics in space (e.g., the Schelling model) (e.g., [9]); and (2) that these geographic distributions may display a degree of alternating map pattern trend because of local social networks that can induce an increasing cancer screening rate when someone in a neighborhood has a positive cancer diagnosis.

## 2. Background

A number of risk factors that are associated with lung cancer incidence have been examined and characterized in the literature (e.g., [10,11,12]); cigarette smoking is the most well-known factor that can trigger lung cancer. Studies also show that nonsmokers exposed to secondhand tobacco have higher risks of developing lung cancer (e.g., [13]). Other life style related risk factors, such as an unhealthy diet and alcohol consumption, increase the risk of developing lung cancer (e.g., [14]). Another suspicious contributor to human lung cancer burden is outdoor air pollution (e.g., fine particulate matter and a concentration of ozone); a number of studies examine, and the findings support, an association between air pollution and lung cancer risk (e.g., [15,16]).

Indicators of socio-economic status also tend to be highly correlated with lung cancer risk. Due to their availability, the use of these variables has been popular amongst researchers to describe lung cancer incidence rates in the literature. For example, Mao et al. [10] report a significant inverse relationship between high socio-economic status, and lung cancer risk. Socio-economic status reflects one’s lifestyle, including diet, working and living conditions, enabling them to be treated as surrogates, and assumed to be associated with lung cancer [17,18]. Specifically, the part of the population with lower socio-economic status (e.g., less educated, below a poverty level, unemployed) tends to have a higher risk of developing lung cancer than their counterparts that are classified with higher socio-economic status [19].

In addition, the risk of developing lung cancer tends to vary across racial/ethnic, age, and sex groups. Alberg et al. [19] argue that lung cancer incidence rates are similar for African and white Americans; however, a higher risk is observed for black American men than white American men. Haiman et al. [20] comment that this difference is attributable to varying smoking behaviors among these ethnic/racial groups. Some case-control studies suggest higher risks of smoking-related lung cancer in women than men (e.g., [21,22]); however, this sex-difference in susceptibility to lung cancer still lacks supporting evidence. Age has been an important risk factor for most of cancers; the risk of lung cancer increases as age increases, seemingly as a part of the natural maturation process. Studies also report that immigration status plays a role in lung cancer risk; for example, United States (U.S.). Asian immigrants have higher lung cancer mortality rates than their U.S.-born counterparts; whereas the rates are lower among U.S. black immigrants than U.S.-born blacks (e.g., [23]), This variation may be attributable to differences in smoking prevalence between the U.S. and the countries of origin, and differences across socio-economic classes (e.g., [24,25]).

Lung cancer incidence rates generally are observed to vary substantially across geographic space. The literature suggest that air pollution is one of the major contributors to this geographic variation [13]. For example, Jacquez and Greiling [26] observe clusters of significantly high lung cancer incidence rates in central Long Island coinciding with a concentration of air toxics. The spatial variation of risk for lung cancer also is attributable to the geographic distribution of population. For example, Kelsall and Diggle [27] report that the prevalence of lung cancer incidence is higher in areas with high social deprivation, which may directly link to smoking behavior and eating habits. A range of spatial models, including Bayesian space-time joint models (e.g., [28]), spatial multilevel regression models (e.g., [29]), and conditional autoregressive models (e.g., [30]), have been applied to account for the geographic variation present in geospatial cancer data analyses.

A RE model frequently is utilized for a longitudinal data analysis exploiting repeated measures over time [31]. For example, it has popularly been applied to model economic/social phenomena. Frondel and Vance [32] specify a RE model to estimate fuel price elasticities with household data. Clarke et al. [33] use a model with both fixed and random effects to analyze the determinants of pupil achievement in primary school, finding that a RE model outperforms a fixed effects only model, based on statistical efficiency. Chen and Tarko [34] employ a RE model to investigate traffic safety in highway work zones, with their results indicating that a RE model furnishes a good option for that type of research.

## 3. Data and Methodology

Lung cancer cases were obtained from the Florida cancer registry. After a data cleaning process that led to removal of duplicates (e.g., patients were diagnosed with lung cancer as a secondary cancer), records containing missing information (e.g., age and sex), and unsuccessfully geocoded records (i.e., failed-to-be-geocoded cases were deleted for the entire state, and then subsets were extracted from the clean dataset for specific study areas), 172,495 cancer incidences were used in data analyses. Cancer points are distributed unevenly across the 67 counties of the state, sample size ranging from 13,918 (Broward County) to 31 (Liberty County), with a median of 1277 (Santa Rosa County). These lung cancer incidences occurred in a 12-year span, from 2000 to 2011. At the block group resolution, a relatively fine geographic resolution, many block groups have zero cancer incidences. In contrast, only 1.98% of the census tracts, a coarser geographic resolution, have zero cancer counts. To avoid the issue of excessive zeros, this research focuses on two geographic resolutions, namely county and census tract, for comparison purpose. In addition, this paper limits its study area to six different metropolitan statistical areas (MSAs) focusing on relatively highly densely populated areas in the state: Pensacola, Tallahassee, Jacksonville, Orlando, Miami, and Tampa.

### 3.1. Lung Cancer Incidence Rates

The crude cancer incidence rate, the ratio of cancer counts and population size at risk, generally is considered as a limited measure because cancer generally occurs at different rates based on age, gender, and even racial group composition of a population. A comparison of crude cancer rates over time or across different geographic areas is likely to be plagued by bias because of different local population compositions [35]. Standardization of disease rates has been proposed to control for changes in population structure. Adjustments of cancer rates for age is a frequently applied standardization [36]. The Centers for Disease Control and Prevention (CDC) also adopts this approach for statistical report purposes. With the availability of age and sex information for lung cancer patients, this research adjusts lung cancer incidence rates for both age and sex.

**Figure 1:**
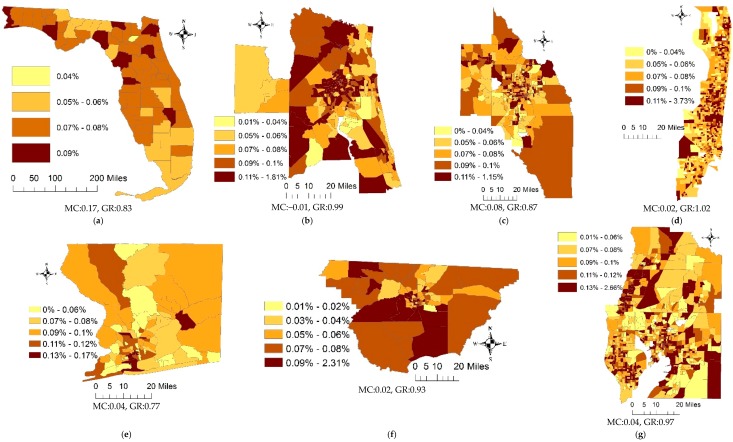
The spatial patterns of adjusted lung cancer incidence rates. (**a**) the State of Florida counties. Census tracts for: (**b**) the Jacksonville MSA; (**c**) the Orlando MSA; (**d**) the Miami MSA; (**e**) the Pensacola MSA; (**f**) the Tallahassee MSA; (**g**) the Tampa MSA.

Figure 1 portrays the geographic distribution of adjusted lung cancer incidence rates across the State of Florida and its six MSAs. The Moran coefficient (MC) and Geary Ratio (GR) statistics suggest adjusted cancer rates exhibit a very weak PSA map pattern at the county resolution (Figure 1a), and random spatial patterns at the census tract resolution (Figure 1b–g).

**Figure A1:**
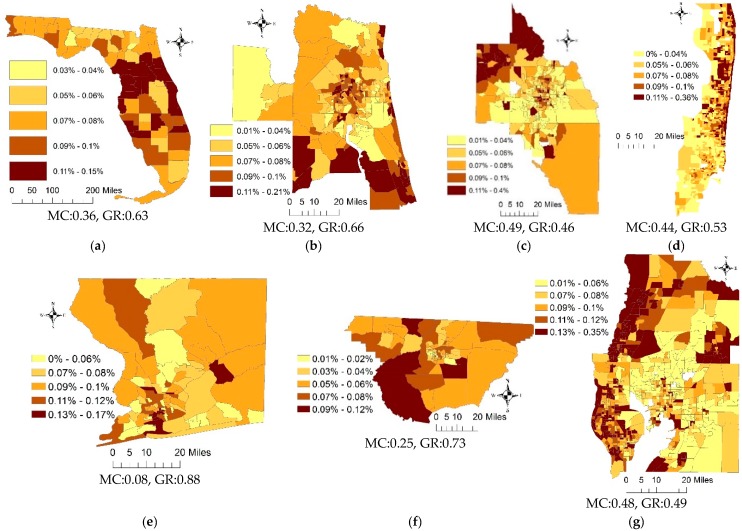
The spatial patterns of crude lung cancer incidence rates. (**a**) the State of Florida counties. Census tracts for: (**b**) the Jacksonville MSA; (**c**) the Orlando MSA; (**d**) the Miami MSA; (**e**) the Pensacola MSA; (**f**) the Tallahassee MSA; (**g**) the Tampa MSA.

Compared with the crude lung cancer incidence rates summarized in the Appendix A (Figure A1), the standardization process tends to reduce spatial clusters of similar cancer rates (i.e., clusters of high values or low values), and generate alternating patterns (i.e., a low lung cancer rate is surrounded by high rates for its neighbors, or a high lung cancer rate is surrounded by low rates for its neighbors) at both the county and census tract resolutions. In addition, due to relatively small populations at the census tract resolution, rate adjustment triggers outliers (e.g., high cancer rates). For example, the highest adjusted cancer rate in the Miami MSA reaches 2.73%, whereas the highest crude rate is 0.36%. Also, more census tracts stand out with high adjusted cancer rates compared with their corresponding crude ones. To mitigate negative impacts of extreme outliers, census tracts with small populations but some cancer counts are aggregated with their neighboring tracts for the analyses summarized in this paper. Specifically, the Miami MSA has 19 such census tracts that were merged into their adjacent tracts; the Tampa and Orlando MSAs have, respectively, five and one such census tracts. Most of these merged census tracts involve commercial, industrial, or coastal land use.

### 3.2. Moran Eigenvector Spatial Filtering

Moran Eigenvector spatial filtering (MESF) is a spatial statistical methodology that introduces a set of eigenvectors into a regression model specification to capture SA. Eigenvectors can be extracted from a transformed spatial weights matrix C, which can be expressed as:

(1) $$\mathrm{MCM}=\left(I-11^{T}/n\right)C(I-11^{T}/n),$$

where **I** is an n-by-n identity matrix, **1** is a n-by-1 vector of ones, n is the number of areal units, and T is the matrix transpose operator. This transformed spatial weights matrix generates n eigenvectors; however, only a subset of them serves as independent variables to be included in a model specification [37]. This subset can be identified from a candidate eigenvector set with a stepwise regression procedure [38].

A RE model can be specified as:

**Y** = **Xβ_X_** + **Z** + **ε**, (2)

where **Y** denotes a response variable, **X** denotes a matrix of covariates, **β_X_** denotes regression coefficients for covariates, **Z** denotes a RE term, and **ε** denotes a regression error term. The RE term, **Z**, is commonly assumed to be normally distributed and uncorrelated with both covariates and residuals, and to have a mean of zero. In order to estimate the RE term and separate it from the residual error **ε**, additional information (e.g., repeated measures furnished in a space-time series, or priors in a Bayesian analysis) are necessary (e.g., [39]). A RE model can be further extended with MESF, in order to accommodate both SSRE and SURE terms simultaneously, as:

**Y** = **Xβ_X_** + **E**_k_**β_E_** + **Z**_SURE_ + **ε**, (3)

where **E**_k_ denotes a subset of eigenvectors, and **β_E_** are unknown coefficients for these eigenvectors. **E**_k_**β_E_** furnishes a SSRE term, and **Z**_SURE_ denotes a SURE term. That is, the RE term, **Z**, is decomposed into the linear combination of **E**_k_**β_E_** and **Z**_SURE_. Furthermore, a separation of the selected eigenvectors, **E**_k_, into PSA and NSA eigenvectors, can furnish a way to investigate PSA and NSA components in a SSRE.

In this paper, space-time lung cancer counts (e.g., *n*-by-*T* = 67-by-12 for the county resolution) furnish the repeated measures for the response variable. The count variable is described with a Poisson probability model by including the logarithmic values of expected lung cancer counts as an offset variable. After a RE term successfully is estimated using the Poisson RE model, a MESF model is specified to estimate the SSRE and SURE components, with the estimated RE term as the independent variable. Essentially, a linear combination of the selected eigenvectors constructs a SSRE term, which is further decomposed into a PSA-NSA mixture [40], and the MESF model residual constitutes the SURE term. Poisson RE and MESF models were implemented in R 3.4.2.; the glmer procedure (package lme4) was utilized to estimate the RE components.

## 4. Results and Discussion

This section summarizes analysis results for both county and census tract resolutions. Regression results for quasi-Poisson and Poisson RE models are compared, and the estimated RE components are portrayed with maps.

### 4.1. The State Scale and County Resolution

Seven variables were retrieved to describe lung cancer incidence rates at the county resolution, including smoking rates from the Florida Department of Health, and socio-economic variables, which are median household income, the percentage of population with a college or higher degree, the percentage of population below a poverty threshold, the percentage of Hispanic population, the percentage of black population, and immigrants, from the U.S. Census Bureau. Table 1 summarizes the estimation results for a Poisson RE model, as well as the results of a quasi-Poisson model for comparison purpose. It shows that the lung cancer data has considerable overdispersion (i.e., excess Poisson variation). However, the extra-Poisson variation successfully is accounted for in the RE model, with the overdispersion parameter decreasing from 13.36 to 2.15. Moreover, an inclusion of the RE term leads to an increase in the pseudo-R^2^, increasing it from 0.30 to 0.74. The VIF values are all less than 10 (e.g., [41,42]), indicating no excessive multi-collinearity among the covariates.

Table 1 also reports standard errors increase in the Poisson RE model specification, which results in significance level changes for some covariates, compared with the results of the covariates-only quasi-Poisson specification. For example, the ratio of population with a college or higher degree, the ratio of population under poverty, and the ratio of black population become insignificant in the RE model. The immigrant variable is included mainly because the State of Florida has gained a large number of immigrants, and papers in the literature argue that lung cancer risk may vary among U.S. residents and immigrants, as discussed in the preceding background. However, the immigrant variable does not have a significant association with lung cancer risk in both models. The only significant variable in the Poisson RE model is the smoking rate, which exhibits a positive relationship with lung cancer risk. The estimated RE term has a mean of zero, and is not correlated with the covariates, as expected.

**Figure 2:**
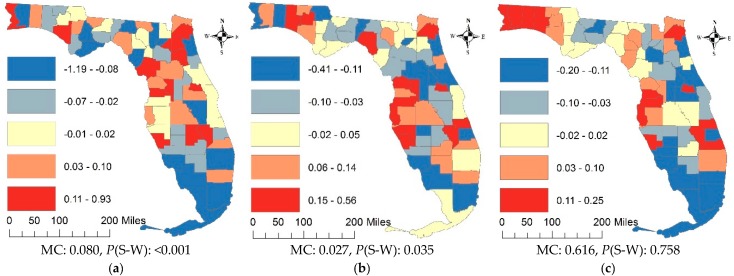

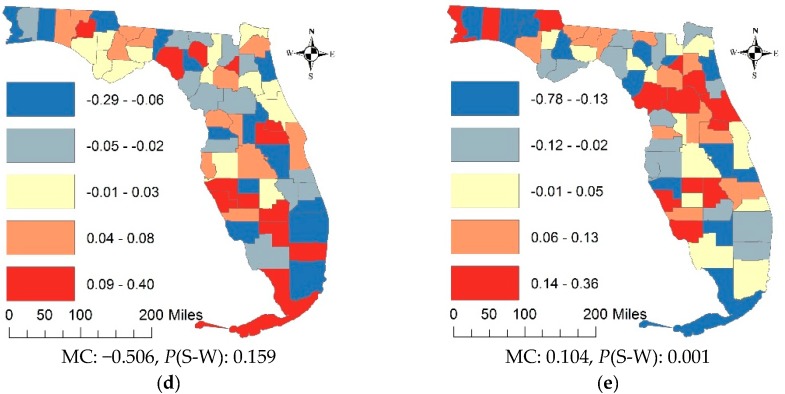
Spatial patterns of RE components for the county resolution. (**a**) the RE term; (**b**) the SSRE term; (**c**) the SSRE-PSA term; (**d**) the SSRE-NSA term; (**e**) the SURE term.

Figure 2 portrays the geographic distributions of RE components at the county resolution. The counties with high/low adjusted lung cancer rates in Figure 1a also are conspicuous in Figure 2a, which captures the major spatial pattern of lung cancer rates. However, the MC values suggest that both the RE and SSRE terms contain trace amounts of SA, which means inclusion of the covariates in the Poisson mixed model explains some degrees of the PSA component observed on Figure 1a. The *p*-values of the Shapiro-Wilk (S-W) normal diagnostic statistic indicate that neither closely conforms to a normal distribution. The decomposition of the SSRE term yields a mixture of moderate-to-strong PSA (Figure 2c) and moderate NSA (Figure 2d). The *p*-values of the S-W statistic indicate that SSRE-PSA and SSRE-NSA are normally distributed. The MC suggests no significant SA in the SURE component, and that it deviates from a bell-shape curve.

### 4.2. The Metropolitan Statistical Area Scale and Census Tract Resolution

Smoking prevalence data are not available at the census tract resolution. So only socio-economic and demographic variables were included to describe lung cancer incidence rates. Results for Poisson RE models for each MSA are compared with covariate-only quasi-Poisson regression results. The overdispersion values larger than one in Table 2 indicate the lung cancer counts are slightly overdispersed for all MSA cases. However, all of them get closer to one for the mixed models. The pseudo-*R*^2^ increases suggest improvements of model performance for all MSAs. A comparison of Table 2 and Table 3 shows that standard errors get larger for the Poisson RE model specifications, which may have an impact on the significance level of independent variables. Including the RE terms also enhances model performance; all RE specifications have larger pseudo-*R*^2^ values.

Table 2 and Table 3 show that median household income is significant in all specifications, and has a negative association with lung cancer risk. Although the well-educated population variable is significant in some cases, exhibiting an inverse relationship, the population below poverty variable tends to be positively associated with lung cancer rates. The relationships between these socio-economic indicators and lung cancer risk corroborates the findings in the literature (e.g., [43,44]). For demographic factors, the estimated results suggest lower lung cancer risks for Hispanics, blacks, and immigrants. Stellman et al. [45] comment that the white and black populations have similar lung cancer risks if their smoking habits are similar. However, studies (e.g., [46]) find that Caucasians are more likely to be heavier smokers than African-American, which makes them more susceptible to lung cancer. Singh and Miller [23] observe that although lung cancer risk varies among different racial/ethical groups, it tends to be lower among U.S. immigrants due to a relatively lower smoking prevalence.

**Figure 3:**
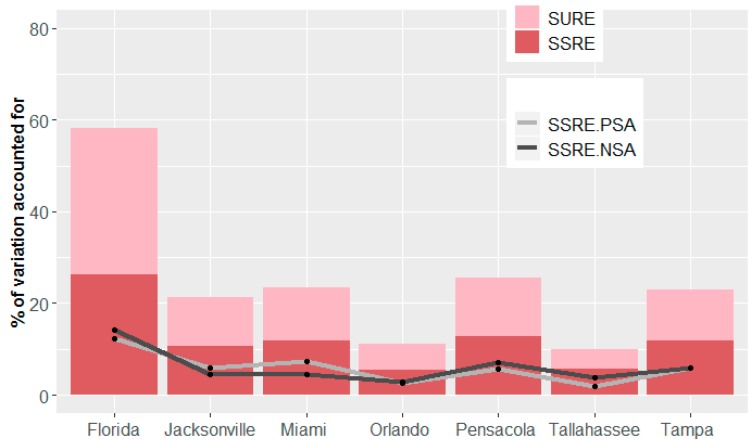
The amount of geographic variation in lung cancer incidence rates accounted for by the RE terms. The first bar is for Florida at the county resolution, and the other six are for the MSAs at the census tract resolution.

Intercept-only RE models are specified for each study area to examine the spatial variation in lung cancer incidence rates. Table 4 summarizes the amount of variation explained by the RE terms. It indicates that the RE terms explain a substantially smaller amount of variations at the census tract resolution than at the county resolution. In addition, this percentage varies across the six MSAs, with the Tallahassee MSA having the lowest statistical explanation (11.64%), and the Pensacola MSA having the highest statistical explanation (27.13%). The average percentage of variation accounted for by the RE terms is roughly 21%, indicating a tremendous amount of unexplained geographic variation in lung cancer rates, particularly at the census tract resolution. Figure 3 depicts the amount of variation accounted for by each RE component beyond that by the covariates. The SSRE and SURE components constituting a RE term explain almost the same amount of variation across all MSAs. Meanwhile, for the two sub-terms of the SSRE, the SSRE-NSA term outperforms the SSRE-PSA term for the Orlando, Pensacola, Tallahassee, and Tampa MSAs.

**Figure 4:**
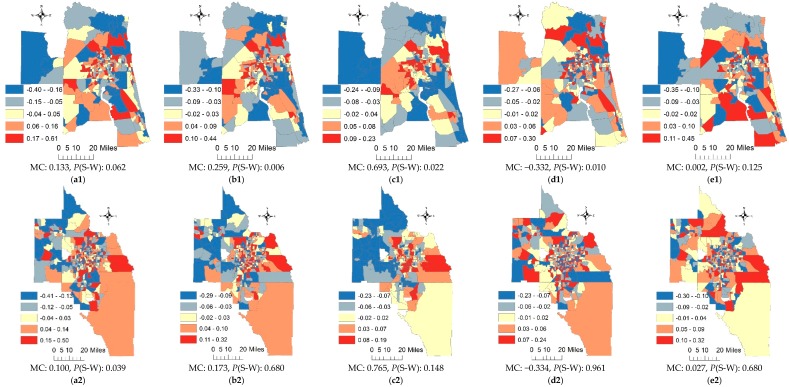

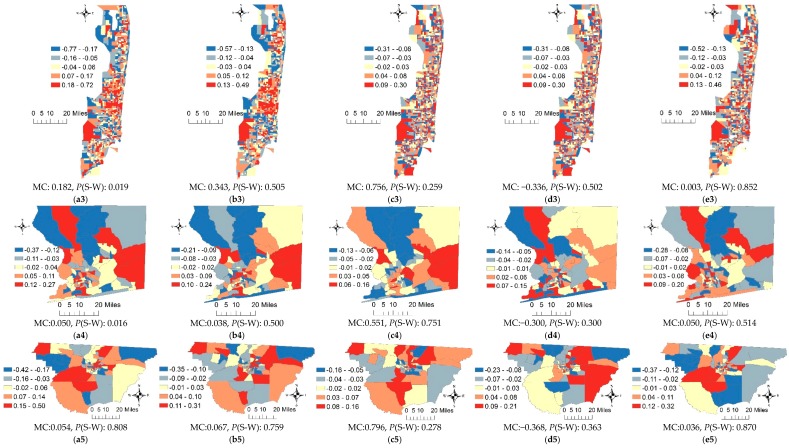

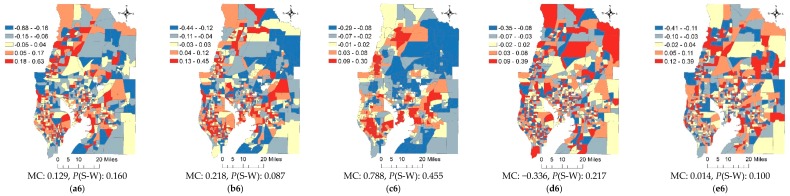
Spatial patterns of RE components at the census tract resolution. (**a1**–**a6**) the RE terms; (**b1**–**b6**) the SSRE terms; (**c1**–**c6**) the SSRE-PSA terms; (**d1**–**d6**) the SSRE-NSA terms; (**e1**–**e6**) the SURE terms. Rows from top to bottom: Jacksonville, Orlando, Miami, Pensacola, Tallahassee, and Tampa MSAs.

Figure 4 portrays the spatial patterns of RE components for the six MSAs. Because the RE components account for relatively low percentages of the geographic variation at the census tract resolution, Figure 4(a1–a6) do not reflect the map patterns of adjusted lung cancer rates well; however, they capture high cancer rates in urban areas, and low rates in rural areas for most of the MSAs, which also are highlighted on their corresponding cancer rates maps. For example, Figure 4(a3) highlights census tracts within Fort Lauderdale and Pompano Beach that have relatively high cancer rates, which also stand out in Figure 1d. The MCs imply a presence of weak PSA in the RE components, except for the Pensacola and Tallahassee MSAs, and the *p*-values of the S-W statistic indicate that they all barely conform to normal distributions.

After a removal of the SURE components from the RE terms, stronger PSA is detected in the SSRE components, with increasing MC values for most MSAs (Figure 4(b1–b6)). However, the SSRE components in the Pensacola and Tallahassee MSAs still exhibit (near-) zero SA. Similarly, a decomposition of these SSRE terms yields mixtures of moderate-to-strong PSA components (Figure 4(c1–c6)) and weak-to-moderate NSA components (Figure 4(d1–d6)) for all MSAs. The *p*-values of the S-W statistic suggest that all of the SSRE-PSA and SSRE-NSA terms closely conform to normal distributions, except for the Jacksonville MSA. Map patterns displayed in Figure 4(e1–e6) appear random, an outcome confirmed by their insignificant MCs. All of the SURE components are normally distributed.

## 5. Conclusions

This research examines the spatial patterns of lung cancer incidence rates at different geographic resolutions and scales in Florida, and also investigates factors that are associated with lung cancer risk. Major findings are as follows. First, lung cancer count data contain a substantial amount of overdispersion (13.36) at the county resolution, whereas they are much less overdispersed (less than 2) at the census tract resolution. A RE model specification successfully addresses this issues. Because the estimated overdispersion parameter is closer to 1 for the RE model specifications, substitution of a negative binomial model becomes unnecessary, which is a desirable outcome given reservations expressed by [47] concerning the suitability of this latter specification for SA situations. Second, a RE model furnishes an efficient method to correct for biased estimation (e.g., underestimated standard errors). Regression results indicate that an inclusion of a RE term, which can serve as a proxy for omitted variables, improves model performance (e.g., it increases pseudo-R^2^ values). Third, estimated results suggest that a risk of lung cancer is positively associated with smoking behavior, and the percentage of population with low socio-economic status (e.g., low household income, poor education), and negatively associated with the percentage of black/Hispanic population, and immigrants. These positive/negative relationships corroborate findings already appearing in the literature.

This research contributes to the literature in the following two ways. First, this research shows that the RE model specifications improve model performance by including a RE terms that successfully accounts for variation beyond that attributable to covariates. Here the RE terms account for 58.39% of the geographic variation in lung cancer incidence rates at the county resolution, and 21% of this variation, on average, at the census tract resolution. This outcome indicates that considerable unexplained variation exists in the lung cancer data at the census tract resolution. This poor statistical explanation probably is attributable to two major factors: aggregating cancer cases into a coarser resolution (e.g., county) averages out noise that present in a finer resolution (e.g., census tract) [48]; and, the massive immigration to Florida of seniors. Generally speaking, population migration over time can contribute to a change in cancer rates, and results in an introduction of a source of variation that is not well described with RE. A purposeful migration for health issues can have a large impact. For example, unhealthy immigrates would choose to move closer to health facilities, or move away from contaminated areas, whereas healthy people relocate to regions that are economically better off (e.g., [49,50]). Immigration, thus, may muddle disease rates in a region with rates increasing in some areas while decreasing in others [51]. In addition, the State of Florida is a well-known destination for retired people. Such movement of elderly people can distort the age pyramid of the state, resulting in an impact on adjusted cancer rates.

Second, the RE term comprises SSRE and SURE components; their MCs indicate the existence of weak-to-moderate PSA (e.g., the Miami MSA) or (near)-zero SA (e.g., the Tallahassee MSA) in the SSRE components. However, a decomposition of the SSRE terms explicitly shows that they essentially are mixtures of moderate-to-strong PSA and weak-to-moderate NSA. Griffith and Arbia [8] utilize a two-SA-parameter spatial simultaneous autoregressive model to uncover a mixture of SA, where the PSA component counterbalances the NSA component. A discovery of SA mixtures has rarely been reported in literature, especially in epidemiology, and its detection can help researchers gain a better understanding of the geographic distribution of, geographic variation of, and risk factors for a disease. As discussed earlier, the moderate-to-strong PSA largely is associated with the geographic distribution of socio-economic phenomenon (e.g., employment status, population migration), whereas the weak-to-moderate NSA likely is linked to mechanisms such as a decrease of lung cancer rates because of increasing cancer screening when lung cancer cases are detected in neighboring places.

This study furnishes motivation for a number of future research efforts. First, a comparison of research outcomes at the county and census tract resolutions reveal a presence of substantial heterogeneity in lung cancer data, and more noise is expected if a spatial analysis is conducted at a finer resolution (e.g., block groups). Thus, extending current research to a finer resolution would be beneficial. Second, a comparison of crude and adjusted lung cancer incidence rates suggests the disappearance of some prominent spatial patterns (e.g., PSA) at both geographic resolutions. However, this observation has rarely been discussed in the literature, and hence a further examination of rate standardization and/or more similar case studies is necessary. Third, to date, the literature about PSA-NSA mixtures is relatively scant. This study only explores the scenario that a weak-to-moderate PSA or (near)-zero SA can be partitioned into a mixture of moderate-to-strong PSA and weak-to-moderate NSA. Other scenarios (e.g., a global strong PSA; moderate NSA) remain to be investigated. Finally, SA mixtures are discovered in the lung cancer data in Florida. Similar research should be conducted to examine if consistent results would be obtained with different empirical data, or for different study areas.

## Figures and Tables

**Table 1:** Estimation results for Poisson models at the county resolution.

Variables	Quasi-Poisson Model				Poisson Random Effects Model			
	Coeff.		Std. Error	VIF	Coeff.		Std. Error	Cor. ^†^
Smoking	4.060	***	0.317	2.158	1.355	*	0.994	<0.001
Income	−0.262		0.262	2.763	0.191		0.617	−0.034
Education	−0.983	*	0.443	4.150	1.116		0.928	<0.001
Poverty	−4.368	***	1.027	7.584	1.608		2.191	<0.001
Hispanic pop	−0.027		0.161	4.738	0.051		0.074	0.074
Black pop	1.587	***	0.284	6.005	−0.627		0.427	0.067
Immigrants	−0.015		0.013	2.449	0.033		0.050	0.021
Overdispersion	13.02				2.12			
Pseudo-R2	0.30				0.75			

Significance codes: *** 0.001, * 0.05, ∙ 0.1. ^†^ This represents correlation coefficients between the RE term and the covariates.

**Table 2:** Estimation results for quasi-Poisson model specifications at the census tract resolution.

Variables	Pensacola MSA				Tallahassee MSA				Jacksonville MSA				Orlando MSA				Miami MSA				Tampa MSA			
	Coeff.		Std. Error	Vif	Coeff.		Std. Error	Vif	Coeff.		Std. Error	Vif	Coeff.		Std. Error	Vif	Coeff.		Std. Error	Vif	Coeff.		Std. Error	Vif
Income	−1.10	***	0.19	3.25	−0.91	**	0.17	2.68	−0.80	***	0.09	2.46	−0.73	***	0.07	1.75	−0.40	***	0.03	1.75	−0.58	***	0.05	1.77
Education	−0.62		0.33	2.80	0.43		0.40	2.32	−0.86	***	0.20	2.56	−0.44	*	0.20	2.47	−0.35	***	0.09	4.00	−0.56	***	0.12	2.29
Poverty	0.85	**	0.31	3.15	0.94	*	0.37	3.15	0.12		0.19	2.66	1.23	***	0.19	2.18	0.01		0.10	2.54	0.54	***	0.11	2.24
Hispanic pop	0.96		0.72	1.14	−0.68		0.54	1.14	0.81	***	0.24	1.11	−0.58	***	0.07	1.39	−0.51	***	0.03	2.22	−0.17	***	0.06	1.44
Black pop	−0.55	***	0.13	2.56	−0.72	***	0.16	2.15	−0.19	***	0.06	2.10	−0.30	***	0.07	1.95	−0.19	***	0.03	1.92	−0.08		0.05	1.50
Immigrants	−6.61	*	3.12	1.16	−2.69		4.29	1.28	−3.07		1.60	1.06	−9.15	***	1.32	1.29	10.21	***	1.29	1.11	−6.06	**	1.86	1.21
Overdispersion	1.08				1.16				1.26				1.22				1.27				1.30			
Pseudo-R^2^	0.14				0.17				0.17				0.40				0.43				0.36			

Significance codes: *** 0.001, ** 0.01, * 0.05, ∙ 0.1.

**Table 3:** Estimation results for Poisson RE model specifications at the census tract resolution.

Variables	Pensacola MSA				Tallahassee MSA				Jacksonville MSA				Orlando MSA				Miami MSA				Tampa MSA			
	Coeff.		Std. Error	Cor. ^†^	Coeff.		Std. Error	Cor. ^†^	Coeff.		Std. Error	Cor. ^†^	Coeff.		Std. Error	Cor. ^†^	Coeff.		Std. Error	Cor. ^†^	Coeff.		Std. Error	Cor. ^†^
Income	−1.06	***	0.27	0.02	−0.99	***	0.23	0.08	−0.78	***	0.12	<0.01	−0.79	***	0.10	−0.02	−0.41	***	0.05	<0.01	−0.65	***	−0.65	−0.04
Education	−0.79	*	0.48	0.01	0.53		0.56	−0.05	−0.86	***	0.31	<0.01	−0.48	*	0.28	0.01	−0.47	***	0.14	<0.01	−0.66	***	−0.66	<0.01
Poverty	0.73		0.46	−0.01	0.91		0.48	−0.07	0.17		0.28	−0.01	1.02	***	0.27	0.01	0.06		0.15	<0.01	0.30	*	0.30	0.05
Hispanic pop	0.44		1.02	0.01	−0.61		0.79	0.10	0.86	***	0.37	<0.01	−0.69	***	0.10	0.01	−0.58	***	0.04	<0.01	−0.17	***	−0.17	−0.02
Black pop	−0.50	***	0.20	−0.01	−0.69	***	0.22	−0.04	−0.17	***	0.09	<0.01	−0.31	***	0.10	<0.01	−0.27	***	0.05	<0.01	−0.03		−0.03	−0.02
Immigrants	−7.82	*	4.52	0.02	−4.91		5.80	−0.05	−2.86		2.58	<0.01	−9.59	***	1.95	<0.01	8.06	***	2.13	<0.01	−9.33	***	−9.33	−0.02
Overdispersion	1.03				1.12				1.10				1.06				1.09				1.07			
Pseudo-*R*^2^	0.19				0.23				0.22				0.44				0.51				0.45			

Significance codes: *** 0.001, * 0.05, ∙ 0.1. ^†^ This represents correlation coefficients between the RE term and covariates.

**Table 4:** The amount of variation accounted for by the RE terms.

Models	Florida	Pensacola MSA	Tallahassee MSA	Jacksonville MSA	Orlando MSA	Miami MSA	Tampa MSA
RE models intercept-only	58.39%	27.13%	11.64%	25.14%	13.68%	24.46%	23.88%
RE models with covariates	58.19%	25.53%	9.91%	21.20%	11.14%	23.47%	22.98%

## References

[1] d’Onofrio A., Mazzetta C., Robertson C., Smans M., Boyle P., Boniol M. (2016). Maps and atlases of cancer mortality: A review of a useful tool to trigger new questions. Ecancermedicalscience.

[2] Wieland S., Cassa C., Mandl K., Berger B. (2008). Revealing the spatial distribution of a disease while preserving privacy. Proc. Natl. Acad. Sci. USA.

[3] Lee M., Chun Y., Griffith D. (2018). An evaluation of kernel smoothing to protect confidentiality of patient locations. Int. J. Urban Sci..

[4] Smith H., Seal S., Sullivan D. (2017). Impact of race, poverty, insurance coverage and resource availability on breast cancer across geographic regions of Mississippi. J. Miss. Acad. Sci..

[5] Roquette R., Nunes B., Painho M. (2018). The relevance of spatial aggregation level and of applied methods in the analysis of geographical distribution of cancer mortality in mainland Portugal (2009–2013). Popul. Health Metr..

[6] Wang N., Mengersen K., Kimlin M., Zhou M., Tong S., Fang L., Wang B., Hu W. (2018). Lung cancer and particulate pollution: A critical review of spatial and temporal analysis evidence. Environ. Res..

[7] Besag J., York J., Mollié A. (1991). Bayesian image restoration, with two applications in spatial statistics. Ann. Inst. Stat. Math..

[8] Griffth D., Arbia G. (2010). Detecting negative spatial autocorrelation in georeferenced random variables. Int. J. Geogr. Inf. Sci..

[9] Fukuda Y., Umezaki M., Nakamura K., Takano T. (2005). Variations in societal characteristics of spatial disease clusters: Examples of colon, lung and breast cancer in Japan. Int. J. Health Geogr..

[10] Mao Y., Hu J., Ugnat A., Semenciw R., Fincham S. (2001). Socioeconomic status and lung cancer risk in Canada. Int. J. Epidemiol..

[11] MacLennan R., Da Costa J., Day N., Law C., Ng Y., Shanmugaratnam K. (1977). Risk factors for lung cancer in Singapore Chinese, a population with high female incidence rates. Int. J. Cancer.

[12] Molina J., Yang P., Cassivi S., Schild S., Adjei A.A. (2008). Non-small cell lung cancer: Epidemiology, risk factors, treatment, and survivorship. Mayo Clinic Proceedings.

[13] Alberg A., Samet J.M. (2003). Epidemiology of lung cancer. Chest.

[14] Feskanich D., Ziegler R., Michaud D., Giovannucci E., Speizer F., Willett W., Colditz G.A. (2000). Prospective study of fruit and vegetable consumption and risk of lung cancer among men and women. J. Natl. Cancer Inst..

[15] Pope C.A., Burnett R., Thun M., Calle E., Krewski D., Ito K., Thurston G.D. (2002). Lung cancer, cardiopulmonary mortality, and long-term exposure to fine particulate air pollution. JAMA.

[16] Vineis P., Forastiere F., Hoek G., Lipsett M. (2004). Outdoor air pollution and lung cancer: Recent epidemiologic evidence. Int. J. Cancer.

[17] Osler M. (1993). Social class and health behavior in Danish adults: A longitudinal study. Public Health.

[18] Pomerleau J., Pederson L., Østbye T., Speechley M., Speechley K.N. (1997). Health behaviours and socio-economic status in Ontario, Canada. Eur. J. Epidemiol..

[19] Alberg A., Brock M., Samet J.M. (2005). Epidemiology of lung cancer: Looking to the future. J. Clin. Oncol..

[20] Haiman C., Stram D., Wilkens L., Pike M., Kolonel L., Henderson B., Le Marchand L. (2006). Ethnic and racial differences in the smoking-related risk of lung cancer. N. Engl. J. Med..

[21] Risch H.A., Howe G.R., Jain M., Burch J.D., Holowaty E.J., Miller A.B. (1993). Are female smokers at higher risk for lung cancer than male smokers? A case-control analysis by histologic type. Am. J. Epidemiol..

[22] Zang E.A., Wynder E.L. (1996). Differences in lung cancer risk between men and women: Examination of the evidence. J. Natl. Cancer Inst..

[23] Singh G., Miller B.A. (2004). Health, life expectancy, and mortality patterns among immigrant populations in the United States. Can. J. Public Health.

[24] Blue L., Fenelon A. (2011). Explaining low mortality among US immigrants relative to native-born Americans: The role of smoking. Int. J. Epidemiol..

[25] Bosdriesz J., Lichthart N., Witvliet M., Busschers W., Stronks K., Kunst A.E. (2013). Smoking prevalence among migrants in the US compared to the US-born and the population in countries of origin. PLoS ONE.

[26] Jacquez G., Greiling D.A. (2003). Geographic boundaries in breast, lung and colorectal cancers in relation to exposure to air toxics in Long Island, New York. Int. J. Health Geogr..

[27] Kelsall J., Diggle P.J. (1998). Spatial variation in risk of disease: A nonparametric binary regression approach. J. R. Stat. Soc. Ser. C Appl. Stat..

[28] Richardson S., Abellan J., Best N. (2006). Bayesian spatio-temporal analysis of joint patterns of male and female lung cancer risks in Yorkshire (UK). Stat. Methods Med. Res..

[29] Jerrett M., Burnett R., Ma R., Pope C.A., Krewski D., Newbold K., Thurston G., Shi Y., Finkelstein N., Calle E.E. (2005). Spatial analysis of air pollution and mortality in Los Angeles. Epidemiology.

[30] Jin X., Carlin B., Banerjee S. (2005). Generalized hierarchical multivariate CAR models for areal data. Biometrics.

[31] Verbeke G., Molenberghs G., Rizopoulos D. (2010). Random effects models for longitudinal data. Longitudinal Research with Latent Variables.

[32] Frondel M., Vance C. (2010). Fixed, random, or something in between. A variant of Hausman’s specification test for panel data estimators. Econ. Lett..

[33] Clarke P., Crawford C., Steele F., Vignoles A.F. (2010). The Choice between Fixed and Random Effects Models: Some Considerations for Educational Research.

[34] Chen E., Tarko A.P. (2014). Modeling safety of highway work zones with random parameters and random effects models. Anal. Methods Accid. Res..

[35] Anderson R., Rosenberg H.M. (1998). Age standardization of death rates: Implementation of the year 2000 standard. Natl. Vital Stat. Rep..

[36] Ahmad O., Boschi-Pinto C., Lopez A., Murray C., Lozano R., Inoue M. (2010). Age Standardization of Rates: A New WHO Standard.

[37] Griffith D.A. (2003). Spatial Autocorrelation and Spatial Filtering: Gaining Understanding through Theory and Scientific Visualization.

[38] Chun Y., Griffith D., Lee M., Sinha P. (2016). Eigenvector selection with stepwise regression techniques to construct eigenvector spatial filters. J. Geogr. Syst..

[39] Griffith D. (2013). Estimating missing data values for georeferenced Poisson counts. Geogr. Anal..

[40] Griffith D.A. (2006). Hidden negative spatial autocorrelation. J. Geogr. Syst..

[41] O’brien R.M. (2007). A caution regarding rules of thumb for variance inflation factors. Qual. Quant..

[42] Craney T., Surles J.G. (2002). Model-dependent variance inflation factor cutoff values. Qual. Eng..

[43] Ward E., Jemal A., Cokkinides V., Singh G., Cardinez C., Ghafoor A., Thun M. (2004). Cancer disparities by race/ethnicity and socioeconomic status. CA Cancer J. Clin..

[44] Clegg L., Reichman M., Miller B., Hankey B., Singh G., Lin Y., Goodman M.T., Lynch C.F., Schwartz S.M., Chen V.W. (2009). Impact of socioeconomic status on cancer incidence and stage at diagnosis: Selected findings from the surveillance, epidemiology, and end results: National Longitudinal Mortality Study. Cancer Causes Control.

[45] Stellman S., Chen Y., Muscat J., Djordjevic I., Richie J.R., Lazarus P., Thompson S., Altorki N., Berwick M., Citron M.L. (2003). Lung cancer risk in white and black Americans. Ann. Epidemiol..

[46] Muscat J., Richie J., Stellman S.D. (2002). Mentholated cigarettes and smoking habits in whites and blacks. Tob. Control.

[47] Diggle P., Milne R.K. (1983). Negative binomial quadrat counts and point processes. Scand. J. Stat..

[48] Openshaw S. (1984). The modifiable areal unit problem. Concepts and Techniques in Modern Geography.

[49] Bentham G. (1988). Migration and morbidity: Implications for geographical studies of disease. Soc. Sci. Med..

[50] Boyle P., Norman P., Rees P. (2002). Does migration exaggerate the relationship between deprivation and limiting long-term illness? A Scottish analysis. Soc. Sci. Med..

[51] Hughes A.E. (2016). Residential Mobility and CRC Screening: A Spatial Analysis of CRC Screening in an Urban Safety-Net Clinic.

